# A Bayesian Logistic Regression Approach to Investigating the Determinants Associated with Never Having Been Screened for Cervical Cancer Amongst Child-Bearing-Age Women in Jordan

**DOI:** 10.3390/ijerph22071000

**Published:** 2025-06-25

**Authors:** Sizwe Vincent Mbona, Anisha Ananth, Retius Chifurira

**Affiliations:** 1 Department of Statistics, Faculty of Applied Sciences, Durban University of Technology, Durban 4001, South Africa; anishas@dut.ac.za; 2 School of Mathematics, Statistics and Computer Science, University of KwaZulu-Natal, Durban 4001, South Africa; chifurira@ukzn.ac.za

**Keywords:** Bayesian modelling, cervical cancer, Jordan, non-informative priors, public health, screening

## Abstract

Cervical cancer continues to be a major global public health problem, with 661,021 estimated new cases and 348,189 deaths reported in 2022. Approximately 53% of women in Jordan reported not being screened for CC in recent years. This study aimed to investigate the determinants associated with not being screened for CC amongst Jordanian women of child-bearing age. This was a cross-sectional study derived from the 2023 Jordanian Demographic Health Survey (JDHS) with 12,580 women aged 15–49 years. The study employed a non-informative Bayesian binary logistic regression approach to identify the factors that are associated with not being screened for CC. Results showed that the prevalence of not being screened for CC was 83.8% (95% CI: 83.3–84.3). The determinants identified in this study were women’s age group (OR = 0.46; 95% CI: 0.34–0.62), education level (OR = 0.56; 95% CI: 0.34–0.91), smoking status (OR = 0.75; 95% CI: 0.63–0.91), women’s nationality (OR = 4.30; 95% CI: 1.03–27.74), breastfeeding status (OR = 1.64; 95% CI: 1.31–2.07), wealth index (OR = 0.61; 95% CI: 0.53–0.71), self-reported health status (OR = 0.74; 95% CI: 0.64–0.87), marital status (OR = 1.45; 95% CI: 1.08–1.96), and HIV testing status (OR = 0.55; 95% CI: 0.40–0.75). The prevalence of not being screened for CC amongst Jordanian women of child-bearing age was found to be very high: a red flag for attention. There is a need for interventions such as community awareness campaigns and education programmes focusing on women younger than 25 years, especially women living in rural and underserved areas. Additionally, incorporating policy interventions into public health facilities and having easy accessibility to tools or screening tests may improve rates of CC screening, and thus reduce the prevalence of CC.

## 1. Introduction

The prevalence and mortality rates from cancer continue to rise globally, particularly as the population ages. This demographic shift is contributing to an increased burden of cancer, including cervical cancer, which disproportionately affects women. Globally, cervical cancer (CC) ranks as the fourth most prevalent gynaecological cancer among women and is also the fourth leading cause of cancer-related deaths in women [1]. Human papillomavirus (HPV), the most common sexually transmitted infection (STI) worldwide, is the primary risk factor significantly associated with CC [2]. Despite the effectiveness of the HPV vaccination and screening, CC remains a significant health problem globally, with an exceptionally high burden in low–middle-income countries due to the inequality of HPV vaccination coverage and access to preventive interventions [3,4,5]. In 2022, an estimated 661,021 new cases of CC were diagnosed worldwide, and 348,189 deaths were reported [6]. In developing and low–middle-income countries, a significant rise in both the incidence and mortality rates of CC has been observed. These countries had a 67.2% 5-year overall survival rate for women diagnosed with CC between 2013 and 2019 [1]. These statistics highlight the alarming global impact of CC, which remains one of the leading causes of cancer-related deaths amongst women, particularly in low–middle-income countries. These high prevalence and mortality rates underscore the urgent need for improved screening, prevention, and early detection programs. There is major health inequality surrounding the utilization of CC screening services globally [7].

Around 90% of newly reported CC cases and deaths globally were in low–middle-income countries [8]. A high proportion of CC cases diagnosed in the early stages were reported to be from developing countries, due to the lack of effective screening programs [9]. Barriers to accessing screening in Jordan included a lack of encouragement from healthcare providers, a preference for female healthcare staff, and limited health education and promotion [10]. Urquhart et al. [11] stated that perceived barriers may include embarrassment, fear, or pain. Screening for CC should occur more frequently than for many other clinical conditions, as it is often oversimplified, for instance, by assuming that a single screening is sufficient or by failing to distinguish between different types of CC in public health messaging. Such oversimplification can result in adverse outcomes, including progression to advanced stages of the disease, as well as increased morbidity and mortality [12,13].

Jordan, like many countries in Asia, is one of the most populous and heavily affected regions in terms of both the incidence and mortality associated with CC. As part of the broader Asian context, which is home to a diverse population with varying access to healthcare, Jordan faces a significant share of the global CC burden [14]. Southeast and south-central Asia have the highest incidence and mortality rates for CC [15,16]. Most Asian countries are using cytology and Visual Inspection with Acetic Acid (VIAA) as the primary methods of screening CC. However, the lack of cytologists, well-trained cytotechnicians and shortages of quality-assured laboratories have hindered the nationwide scale-up of cytology [2,17]. Liu et al. [18] revealed that the screening of CC in most of Asian countries is via outreach clinics. However, they often miss the target population, leading to low rates of screening. The major challenges faced by most Asian countries include health facilities and a lack of resources and trained professionals [17,19,20].

The lack of screening is a major obstacle in the prevention and early detection of CC. Without regular screening, many cases of CC go undiagnosed until they reach advanced stages, when treatment options are more limited and survival rates are lower. Almost 53% of women in Jordan reported not being screened for CC in recent years [21,22]. Roughly 3.63 million women aged 15 years and older are at risk of CC [23]. Additionally, around 115 women are diagnosed with CC annually, with 71 succumbing to the disease each year. In 2023, the age-standardized incidence rate of CC was 2.91 per 100,000 Jordanian women [23]. A qualitative study by Srinath et al. [24], which investigated CC screening, concluded that the significant barriers to efficient screening include the fear of cancer diagnosis; the lack of comfort at healthcare centres; higher service costs; the discourtesy of healthcare staff towards patients; and a lack of available and accessible healthcare quality services in Jordan and developed countries.

Although HPV is the primary cause of CC, other risk factors like starting sexual activity at an age younger than 21 years; a history of sexually transmitted disease; smoking; five years or more of hormonal contraceptive use; multiple sexual partners; and three or more full-term pregnancies (high parity) have been identified as significant factors associated with CC [25]. Age, marital status, education level, employment status, and income level are socio-demographic factors that have also been found to be significantly associated with CC screening by previous researchers [26,27].

The rate of CC screening in Jordan remains inadequate due to the absence of a dedicated national screening program. Additionally, there is a lack of studies focusing on the awareness and knowledge of smear tests among Jordanian women [28,29]. Therefore, this study’s aim was to investigate the determinants associated with never having been screened for CC amongst Jordanian women aged 15–49 years using data extracted from the 2023 Demographic Health Survey in Jordan.

Despite the growing recognition of Bayesian methods in health research, their application in investigating the determinants of never having being screened for cervical cancer remains limited. Most existing studies rely on traditional frequentist approaches, which may not fully account for uncertainty or incorporate prior knowledge. This gap highlights the need for more Bayesian-based analyses to enhance the robustness and interpretability of findings in cervical cancer screening research. This paper applied a Bayesian logistic regression approach. To the researchers’ best knowledge, this kind of study has never been conducted previously.

Understanding the risk factors that affect Jordanian child-bearing-aged women seeking screening for CC would play a significant role in designing and implementing effective screening programs. The results and recommendations from this study would be of critical value in assisting policymakers and public health practitioners to establish a screening policy tailored to women’s needs and would thus help to reduce the burden of the disease [30]. Different strategies will be of assistance to the Jordanian community, health planners, and government and policymakers to increase awareness campaigns, create a comprehensive health education programme for the community, and make cancer screening centres easily accessible to everyone, which may best reach women who have never been screened for CC.

## 2. Methods

### 2.1. Data Source

This study used secondary data extracted from the 2023 Jordan Demographic Health Survey (JDHS). The survey sample was designed to produce representative results for the country, for urban and rural areas, for the country’s three regions (Central, North, and South) and 12 governorates, and for three national groups (Jordanians, Syrians, and individuals of other nationalities). JDHS 2023 is the most recent data collected on CC screening amongst women of reproductive age. Data collection took place between January and June 2023. The sample for the 2023 JDHS was a stratified sample selected in two stages from the 2015 census frame. Stratification was achieved by separating each governorate into urban and rural areas. A total of 26 sampling strata were constructed. Samples were selected independently in each sampling stratum through a two-stage selection process, according to the sample allocation.

Firstly, 970 clusters were selected with probability proportional to cluster size. A household listing operation was carried out in all the selected sample clusters, and the resulting lists of households served as the sampling frame for the selection of households in the next stage. In the second stage of selection, a fixed number of 20 households per cluster were selected with an equal probability systematic selection from the newly created household listing. No replacements and no changes in pre-selected households were allowed in the implementation stages to avoid bias. Eligibility for inclusion was determined by the presence of women aged 15–49 years in the sampled household the night before the interview, as this age group encompasses the reproductive years. Women who were in hospitals, prisons, hotels, barracks, and camps were excluded. A total of 20,054 households were selected for the sample, 19,809 of which were occupied. Of the occupied households, 19,475 were successfully interviewed, yielding a response rate of 98%. In the interviewed households, 13,020 eligible women aged 15–49 years were identified for individual interviews; interviews were completed with 12,595 women, yielding a response rate of 97%.

The outcome variable in this study was cervical cancer screening, which was measured by the question “ever tested for cervical cancer by healthcare provider?” The binary outcome was categorized as yes/no, consistent with the methodology used by other researchers who analysed DHS data [31,32,33]. Women who were uncertain about their screening status were excluded from the study, resulting in a final sample of 12,580 women.

The risk factors or predictors of cervical cancer were age in years; educational level (no education, primary, secondary, higher); smoking status (non-smoker, smoker); residence type (urban, rural); nationality (Jordanian, Egyptian, Syrian, Iraqui, other Arab nationalities, non-Arab nationalities); currently breastfeeding (no, yes); wealth index (poorest, poor, middle, richer, richest); self-reported health status (very good, good, moderate, bad, very bad); health insurance coverage (yes, no); marital status (single, married, living with partner, widowed, divorced, separated/no longer living together); work status (not working, working); and ever having been tested for HIV (no, yes). These variables were selected for this study because some were found to have a significant effect on CC [32,34], while the others were chosen by the researchers based on their potential to influence CC outcomes.

The age of women was recorded as a continuous variable and then grouped into three categories, 15–24, 25–34, and 35–49 years, following the approach of previous researchers [31,32,34]. The wealth index was also re-categorized from five to three categories: ‘poor’ (combining poorest and poorer), ‘middle’ (middle wealth), and ‘rich’ (combining richer and richest) [31,32,34,35]. Additionally, self-reported health status was reclassified from five to three categories: ‘good’ (combining very good and good), ‘moderate’ (moderate health), and ‘bad’ (combining bad and very bad).

Multiple Imputation by Chained Equations (MICE) is a useful method for addressing missing data. Although the researchers would have applied MICE to impute missing values, the dataset contained no missing data. Additionally, multicollinearity was assessed using inter-correlation analysis, and no evidence of multicollinearity was found.

### 2.2. Ethical Consideration

The researchers of this study obtained written permission from the Demographic Health Survey program to download at the following link: https://www.dhsprogram.com (accessed on 21 August 2024) and use the data. The questionnaire for standard DHS was reviewed and approved by the Medical Research Council, Health Research Institute, and ICF’s Internal Review Board (IRB).

### 2.3. Statistical Analysis

In many fields of study, including medicine, business, and social sciences, the application of logistic regression to binary or categorical outcomes data has been extensively used and regarded as the standard approach in the past decades. This classical approach is simple to understand, easy to compute, and the regression coefficients have simple interpretations in terms of odds ratios (OR) [36,37]. The logistic regression model is mostly applied in cross-sectional and case–control studies to estimate OR through the maximum likelihood estimation (MLE) method [38,39]. The inference is made by maximum likelihood due to the non-linearity of the model. Despite the widespread use of the classical logistic regression model because of its simplicity, the MLE encounters significant bias for small samples [40,41]. The Bayesian logistic regression (BLR) model has an advantage over MLE in terms of estimating model parameters based on their posterior distribution, which is a combination of prior distribution (data from personal experience or from previous studies) and the likelihood function (observed data) [42]. Moreover, BLR can model DHS data, which involves complex survey designs and can handle missing values through imputation directly in the model.

A comprehensive description of the methodological procedures for both Classical and Bayesian logistic regression analyses is provided in Supplementary Material A, offering readers the technical insights necessary for a deeper understanding of the analytical framework used in this study. All statistical analyses were conducted using R statistical software (version 4.4.2). Bayesian logistic regression was carried out using *brms* through the *rstan* package. A *p*-value less than 0.05 was considered indicative of statistical significance.

## 3. Results

Amongst 12,580 participants in this study, the weighted mean age was 37 years (standard deviation = 8.31), with a few (8.6%) respondents aged between 15 and 24 years, and more than half (57.0%) having a secondary level of education. Most women were non-smokers (91.6%), resided in urban areas (91.1%), and most of them were Jordanian (88.6%). The researchers observed that 90.6% of the respondents were not breastfeeding. Regarding wealth status, 40.5% of women were from poor households, followed by those who were rich (34.1%), and 21.4% who were in the middle wealth index. Of all respondents, 85.7% reported having a good health status, 12.9% having moderate health, and 1.4% having a bad health status. More than half (69.0%) of women who participated in this study reported that they are covered by health insurance. No women were single or living with a partner in this current study. It was observed that 92.3% of women were married, with a small percentage of widowed women (2.8%), divorcees (4.8%), and those who were no longer living with their partner/separated (0.1%). Most women were not working (86.4%). Furthermore, almost all (97.8) women in this study had never been tested for HIV (Table 1).

The overall weighted prevalence of never having been screened for CC was 83.8% and was highest amongst women aged 35–49 years (56.1%), with secondary education (57.8%), women who were not smoking (92.6%), and women residing in urban areas (90.6%). Similarly, the prevalence of never having been screened for CC was highest amongst Jordanian women (87.7%), not breastfeeding (89.6%), coming from poor households (43.8%), with good health status (86.3%), not working (87.1%), and those who never tested for HIV (98.1%) (Table 1).

A Bayesian logistic regression analysis was employed to make inferences about the parameters of the model. Parameter estimation was carried out using the Markov Chain Monte Carlo (MCMC) via the Metropolis-Hastings Algorithm. Convergence was reached after a burn-in period of 60,000 iterations. The estimated means, posterior standard errors, and odds ratios with 95% credible intervals are presented in Table 2. Many covariates included in this study were significant, indicating the association with the prevalence of never having been tested for CC. This study’s results show that the odds of never having been screened for CC were 0.46 times lower for the age group 25–34 years (OR = 0.46; 95% CI: 0.34–0.62) and 0.27 times lower for the age group 35–49 years (OR = 0.27; 95% CI: 0.20–0.36), compared to those in the age group 15–24 years. Women with a primary level of education had lower odds of never having been screened for CC (OR = 0.56; 95% CI: 0.34–0.91), followed by women with a secondary level of education (OR = 0.43; 95% CI: 0.27–0.66) and higher education (OR = 0.45; 95% CI: 0.28–0.69), compared to women with no education.

The results showed that smoking women are at 25% reduced odds (OR = 0.75; 95% CI: 0.63–0.91) of never having been screened for CC compared to those who are non-smokers. The odds of never having been screened for CC in Syrian women were 1.55 times higher (OR = 1.55; 95% CI: 1.26–1.90) compared to Jordanian women. Furthermore, the odds of ‘never screened for CC’ in women who are not Arab nationalities were 4.30 times higher compared to Jordanian women (OR = 4.30; 95% CI: 1.03–27.74). Breastfeeding women had 64% higher odds (OR = 1.64; 95% CI: 1.31–2.07) of never having been screened for CC compared to women who were not breastfeeding. Women from the middle wealth index (OR = 0.61; 95% CI: 0.53–0.71) and those coming from rich households (OR = 0.42; 95% CI: 0.37–0.48) had lower odds of ‘never screened for CC’ compared to women coming from poor households.

Self-reported health status was a significant factor affecting CC screening in Jordanian women. It was observed that a woman who reported moderate health status was 0.74 times less likely to never screen for CC (OR = 0.74; 95% CI: 0.64–0.87) than a woman with good health status. The findings also showed that a woman with bad health status was 0.46 (OR = 0.46; 95% CI: 0.32–0.67) times less likely to never screen for CC than a woman with good health status. Divorced women were 1.45 times more likely to never screen for CC compared to those who were married (OR = 1.45; 95% CI: 1.08–1.96). Furthermore, women who once tested for HIV were 0.55 times less likely to never screen for CC (OR = 0.55; 95% CI: 0.40–0.75) compared to those who had never tested for HIV.

The independent variables that are not reported here but that are depicted in Table 2 were not statistically significant since their 95% credible intervals included 1. In this study, based on the results obtained, the researchers concluded that age, education level, smoking status, nationality, breastfeeding status, wealth index, health status, marital status, and HIV test are significantly associated with ‘never screened for CC’.

The convergence of the MCMC sampling algorithm was checked by density plots and time-series (history) plots (see Supplementary Material B). The left panel of Figure S1 in Supplementary Material B displays the density plots of the regression coefficients included in the model. It was observed that the densities of the posterior distributions converged under the Gaussian prior distribution used. Furthermore, the MCMC time-series plots (right panels) illustrate how well the samples are mixing. They all look like a horizontal band, with no long downward or upward trends, indicating that the chains have converged and are providing a good understanding of the estimated coefficients from the Bayesian regression model. Therefore, one can conclude that the Bayesian inference was successful. This was also confirmed with the high accuracy (86.4%) obtained from the Bayesian logistic regression under Gaussian previously (see Table 2).

## 4. Discussion

Despite the increase in implementation of screening protocols, community awareness, and the HPV vaccination, cervical cancer continues to be among the most common global health problems in women, with approximately 660,000 new cases and 350,000 deaths in 2022 [43]. This cross-sectional study employed a Bayesian multivariable logistic regression approach, which showed a high accuracy of approximately 86.4%, to investigate determinants associated with ‘never screened for CC’ amongst women in Jordan. The mean age of the Jordanian women presenting for screening in this study was 37 years, and more than half (59.5%) were between 34 and 49 years old. The results of this study showed a significant prevalence of never having been screened for CC. The prevalence rate of never having been screened for CC was 83.8% (95% CI: 83.3–84.3) among our study population. This finding is in line with other studies [44,45,46]. This might be due to the lack of awareness programmes, access to screening services, or availability of the services in health facilities. Furthermore, the high burden on healthcare providers might be a factor which may increase the rate of never having been screened for CC.

A study conducted by Fram et al. [22] used a sample of 655 Jordanian women and found that 51.9% of women reported having no idea about the Pap smear test, which is regarded as one of the most reliable methods for diagnosing CC [29,47]. A study in China revealed that women with less knowledge about screening tests were less likely to have a Pap smear test performed [48]. Additionally, several factors can reduce the rate of CC screening, such as discomfort with male healthcare providers, spousal disapproval, and fear of a speculum examination [49].

The determinants identified as significant predictors of ‘never screened for CC’ were women’s age group, education level, smoking status, women nationality, breastfeeding status, wealth index, self-reported health status, marital status, and HIV testing status. This study found that women aged 25–34 years had lower odds of never having been screened for CC than those aged 15–24 years, followed by those aged 35–49 years, compared to those aged 15–24 years. Other studies found that the odds of CC screening increased with age [44,50,51,52,53]. This might be because the symptoms of CC present at later ages, above 30 years old. Globally, the average age of CC diagnosis is 50 years, with most women being diagnosed between 35 and 44 years [54]. Another possibility could be that there is a lack of public awareness and clinical guidelines of CC screening due to the scarcity of resources. Therefore, governments and other organisations should create programmes that target younger women in order to improve the screening rate. The odds of ‘never screened for CC’ amongst women with education was lower compared to uneducated women. Similar findings are observed in other studies, as screening for CC decreases with a lack of formal education [50,55]. Awareness of CC screening in Jordan should target young women with less or no education.

The other determinant of not screening for CC was smoking status. The odds for women who smoke cigarettes were 0.75 times lower for never having been screened for CC as compared to non-smokers. A study by Eng et al. [56] found that former smokers were more likely to be screened for CC than those who never smoke cigarettes. Healthcare practitioners could pay more attention to those population groups. The odds of never having been screened for CC were 1.55 times higher amongst Syrian women compared to Jordanian women, and 4.30 times higher amongst women who are not of Arab nationality compared to Jordanian women. The possible reason could be that women who are not Jordanian have no knowledge of where to find screening centres. In this study, it was also found that the odds of ‘never screened for CC’ were 1.64 times higher in breastfeeding women compared to those who are not breastfeeding. The possible explanation for this could be that breastfeeding women find it difficult to go long distances with a baby to seek screening. Another reason could be that breastfeeding women normally undergo other tests during their pregnancies and they may think they have a healthier lifestyle.

Women in the middle- and high-income groups are 39% and 58% less likely, respectively, to have never undergone CC screening compared to those in the low-income group. This finding aligns with previous studies conducted in African countries [50,52,57]. The low rate of CC screening among women in the low-income group may be attributed to limited access to healthcare services, as they often live in areas far from public health facilities and face financial barriers to accessing care. The study also found that marital status was significantly associated with ‘never screened for CC’. The odds of never having been screened for CC were 1.45 times higher in divorced women compared to married women. Similar findings were observed in other studies, where there was an increased uptake of CC screening amongst married women compared to unmarried women [58,59]. This study found that CC screening decreases with an increase in testing for HIV. The odds of ‘never screened for CC’ were 0.55 times lower in women who had ever been tested for HIV compared to women who had never been tested for HIV. This could be due to the fact that once a woman receives results that she is HIV-positive, she becomes afraid of screening for CC because results might also come back positive.

## 5. Strengths and Limitations

The current study used recent Jordan demographic Health Survey data, which are wide-ranging, suggesting that these results can be generalised nationally. In addition, the study considered both the individual and community-level variables with appropriate methods of analysis. However, this study had some limitations. It was limited because of its cross-sectional design, and thus causality cannot be inferred. The study was limited to only women aged between 15 and 49 years in Jordan. This study focused only on the prevalence and determinants of ‘never screened for CC’. Furthermore, since the study was focused only on women in Jordan, findings may not be generalised to other countries. The researchers used secondary data, and some variables like beliefs, culture, and behaviour of women were not assessed.

## 6. Conclusions and Recommendations

The prevalence of never having been screened for CC amongst Jordanian women was found to be alarmingly high, signalling a critical need for immediate attention and action. This underlines the urgency for targeted interventions that address the barriers preventing women from accessing screening services. Community awareness campaigns and educational programs are essential, particularly for younger women under 25 years, who are at higher risk of being overlooked in current healthcare initiatives. Special focus should be placed on reaching women living in rural and underserved areas, where access to healthcare services and knowledge about CC screening are often limited.

Furthermore, policy interventions are crucial in improving the accessibility and affordability of CC screening. Public health facilities should be equipped with the necessary tools and resources to offer these tests, and efforts should be made to streamline the process, making it more convenient and less intimidating for women to seek screening. By enhancing the accessibility of screening programs and integrating them into routine healthcare practices, one can significantly increase the rate of CC screening, thereby reducing the incidence and prevalence of CC in Jordan. Long-term efforts should aim to build a comprehensive, supportive healthcare infrastructure that prioritizes prevention and early detection, ultimately saving lives and improving public health outcomes.

## Figures and Tables

**Table 1:** Socio-demographic characteristics of the study respondents (n = 12,580).

Characteristic	Category	Weighted Total	Weighted Prevalence
		n (%)	% (95% CI)
Age group (years)	15–24	1084 (8.6)	9.9 (9.4–10.4)
	25–34	4016 (31.9)	34.0 (33.2–34.8)
	35–49	7485 (59.5)	56.1 (55.3–56.9)
Education level	No education	268 (2.1)	2.4 (2.2–2.6)
	Primary	783 (6.2)	6.8 (6.4–7.2)
	Secondary	7178 (57.0)	57.8 (57.0–58.6)
	Higher	4357 (34.6)	33.1 (32.3–33.9)
Smoking status	Non-smoker	11,526 (91.6)	92.6 (92.2–93.0)
	Smoker	1060 (8.4)	7.4 (7.0–7.8)
Residence type	Urban	11,468 (91.1)	90.6 (90.1–91.1)
	Rural	1117 (8.9)	9.4 (8.9–9.9)
Nationality	Jordanian	11,145 (88.6)	9246 (87.7)
	Egyptian	61 (0.5)	54 (0.5)
	Syrian	979 (7.8)	891 (8.4)
	Iraqui	69 (0.5)	60 (0.6)
	Other Arab nationalities	275 (2.2)	241 (2.3)
	Not Arab nationalities	56 (0.4)	55 (0.5)
Currently breastfeeding	No	11,401 (90.6)	9451 (89.6)
	Yes	1184 (9.4)	1096 (10.4)
Wealth status	Poor	5097 (40.5)	4618 (43.8)
	Middle	2688 (21.4)	2247 (21.3)
	Rich	4801 (34.1)	3682 (34.9)
Self-reported health status	Good	10,790 (85.7)	9106 (86.3)
	Moderate	1621 (12.9)	1316 (12.5)
	Bad	174 (1.4)	125 (1.2)
Covered by health insurance	No	3904 (31.0)	3253 (30.8)
	Yes	8682 (69.0)	7294 (69.2)
Marital status	Single	-	-
	Married	11,615 (92.3)	9705 (92.0)
	Living with partner	-	-
	Widowed	358 (2.8)	311 (2.9)
	Divorced	601 (4.8)	521 (4.9)
	No longer living together/separated	12 (0.1)	11 (0.1)
Employment status	Not working	10,872 (86.4)	9190 (87.1)
	Working	1715 (13.6)	1358 (12.9)
Ever been tested for HIV	No	12,313 (97.8)	10,348 (98.1)
	Yes	272 (2.2)	199 (1.9)

n = Number; % = percentage; CI = confidence interval.

**Table 2:** Posterior summary statistics from the Bayesian logistic regression model.

Covariate	Posterior Mean	Posterior Standard Error	OR (95% CI)
Intercept	3.96	1.33	52.43 (30.55–93.59) *
**Age group** (Ref: 15–24)			
25–34	−0.78	1.17	0.46 (0.34–0.62) *
35–49	−1.32	1.16	0.27 (0.20–0.36) *
**Education level** (Ref: No education)			
Primary	−0.58	1.29	0.56 (0.34–0.91) *
Secondary	−0.84	1.26	0.43 (0.27–0.66) *
Higher	−0.81	1.27	0.45 (0.28–0.69) *
**Smoking status** (Ref: Non-smoker)			
Smoke	−0.28	1.10	0.75 (0.63–0.91) *
**Residence type** (Ref: Urban)			
Rural	0.10	1.08	1.11 (0.96–1.28)
**Nationality** (Ref: Jordanian)			
Egyptian	0.28	1.51	1.33 (0.63–3.22)
Syrian	0.44	1.11	1.55 (1.26–1.90) *
Iraqui	0.88	1.94	2.42 (0.76–10.93)
Other Arab nationalities	0.23	1.21	1.26 (0.89–1.86)
Not Arab nationalities	1.46	2.30	4.30 (1.03–27.74) *
**Currently breastfeeding** (Ref: No)			
Yes	0.50	1.12	1.64 (1.31–2.07) *
**Wealth status** (Ref: Poor)			
Middle	−0.50	1.08	0.61 (0.53–0.71) *
Rich	−0.87	1.07	0.42 (0.37–0.48) *
**Self-reported health status** (Ref: Good)			
Moderate	−0.30	1.08	0.74 (0.64–0.87) *
Bad	−0.78	1.22	0.46 (0.32–0.67) *
**Covered by health insurance** (Ref: No)			
Yes	0.14	1.07	1.15 (1.00–1.31)
**Marital status** (Ref: Married)			
Widowed	0.22	1.17	1.25 (0.92–1.73)
Divorced	0.37	1.17	1.45 (1.08–1.96) *
No longer living together/separated	0.59	2.44	1.80 (0.41–13.07)
**Employment status** (Ref: Not working)			
Working	0.09	1.08	1.10 (0.93–1.28)
**Ever been tested for HIV** (Ref: No)			
Yes	−0.60	1.18	0.55 (0.40–0.75) *
Accuracy		0.864	

OR = Odds ratio; CI = credible interval for odds ratio; * Statistically significant

## Data Availability

The dataset generated and analysed during the current study is publicly available from the DHS team https://dhsprogram.com/ (accessed on 21 August 2024) upon request.

## Supplementary Materials

The following supporting information can be downloaded at: https://www.mdpi.com/article/10.3390/ijerph22071000/s1. Reference [60] are cited in the supplementary materials.
