# Supplementary material for: A Bayesian Logistic Regression Approach to Investigating the Determinants Associated with Never Having Been Screened for Cervical Cancer Amongst Child-Bearing-Age Women in Jordan
Source: Int J Environ Res Public Health. 2025 Jun 25;22(7):1000. doi: 10.3390/ijerph22071000 (PMC12295522; doi:10.3390/ijerph22071000)
Supplement: Supplementary file 1 [file ijerph-22-01000-s001.zip › ijerph-3715325-supplementary.pdf]

Supplementary Materials A

The Standard (frequentist) Logistic Regression model

In many cases, the classical logistic regression usually models the relationship between the binary outcome (response) variable with the predictor variables, either categorical or continuous. The logistic regression is simpler because only the regression parameters are estimated, and no variance term is to be estimated. Suppose that the outcome variable y is binary with the probability of success p and probability of failure $q = 1 - p$, then logistic regression model is defined as:

$$logit\,(p) = \log\left(\frac{p}{1-p}\right) = \beta_0 + \beta_1 x_1 + \beta_2 x_2 + \cdots + \beta_p x_p \qquad (1)$$

where $x_1, x_2, \dots, x_p$ are p predictor variables, β_0 is the intercept, and $\beta_1, \beta_2, \dots, \beta_p$ are the unknown regression parameters to be estimated. The predicted value of y_i $(i = 1, 2, \dots, n)$ for the n independent Bernoulli experiment with the probability of success $P(y_i = 1)$ given by:

$$P(y_i = 1) = \frac{exp(\beta_0 + \beta_1 x_{1i} + \beta_2 x_{2i} + \cdots + \beta_p x_{pi})}{1 + exp(\beta_0 + \beta_1 x_{1i} + \beta_2 x_{2i} + \cdots + \beta_p x_{pi})} \qquad (2)$$

where y_i indicates the presence, $y_i = 1$, or absence $y_i = 0$ of the event for that subject i.

The hypothesis used in this test is:

H_0: $\beta_0 = 0$

H_1: $\beta_j \neq 0$ where $j = 1, 2, \dots, p$

that is, to determine whether a predictor variable has a significant effect on the outcome variable or not. The Wald test statistics is:

$$W = \frac{\beta_0}{se\,(\beta_j)}, \qquad [W \sim N(0,1)] \qquad (3)$$

The null hypothesis (H_0) is rejected if the test statistic value $|W| > Z$, and that concludes that the predictor variable significantly affects the outcome variable.

Bayesian Logistic Regression (BLR)

The BLR model describes inference with emphasis on the prior distribution, likelihood function and posterior distribution. The effect size of each parameter is estimated from the posterior distribution, which is the distribution of both the prior information and the likelihood of the data. This is done by multiplying the data likelihood function and the prior distribution for the parameter estimates to obtain the posterior distribution, and then all parameter estimates are drawn from it. The three components are formally combined by Bayes' rule as:

$$Posterior\ distribution\ =\ likelihood\ function \times prior\ distribution \tag{4}$$

The posterior distribution contains all the information about the parameters in the model. This is shown in (4), that the information contained in the sample (likelihood function) is combined with the information from other sources (prior distribution) to obtain the posterior distribution.

Likelihood Function

The likelihood function used by Bayesian draws from frequentist inference. Given the probability of success (which in logistic regression varies from one subject to another, depending on their covariates), the likelihood contribution from the ith subject is binomial:

$$likelihood_i = [\pi(x_i)]^{y_i}[1-\pi(x_i)]^{1-y_i} \tag{5}$$

In (5) above, $\pi(x_i)$ is the probability of the event for subject i that has covariate vector x_i. Again, in the classical logistic regression, $\pi(x_i)$ is given by:

$$\pi(x) = \frac{exp\left(\beta_0 + \beta_1 x_1 + \beta_2 x_2 + \cdots + \beta_p x_p\right)}{1 + exp\left(\beta_0 + \beta_1 x_1 + \beta_2 x_2 + \cdots + \beta_p x_p\right)} \tag{6}$$

Now, the likelihood contribution from the ith subject is:

$$likelihood_i = \left[\frac{exp(\beta_0+\beta_1 x_{1i}+\beta_2 x_{2i}+\cdots+\beta_p x_{pi})}{1+exp\left(\beta_0+\beta_1 x_{1i}+\beta_2 x_{2i}+\cdots+\beta_p x_{pi}\right)}\right]^{y_i}\left[1 - \frac{exp(\beta_0+\beta_1 x_{1i}+\beta_2 x_{2i}+\cdots+\beta_p x_{pi})}{1+exp\left(\beta_0+\beta_1 x_{1i}+\beta_2 x_{2i}+\cdots+\beta_p x_{pi}\right)}\right]^{1-y_i} \tag{7}$$

Since subjects are assumed to be independent of each other, the likelihood function for n subjects is given by:

$$likelihood = \prod_{i=1}^{n} \left\{ \left[\frac{exp(\beta_0+\beta_1 x_{1i}+\beta_2 x_{2i}+\cdots+\beta_p x_{pi})}{1+exp(\beta_0+\beta_1 x_{1i}+\beta_2 x_{2i}+\cdots+\beta_p x_{pi})} \right]^{y_i} \left[1 - \frac{exp(\beta_0+\beta_1 x_{1i}+\beta_2 x_{2i}+\cdots+\beta_p x_{pi})}{1+exp(\beta_0+\beta_1 x_{1i}+\beta_2 x_{2i}+\cdots+\beta_p x_{pi})} \right]^{1-y_i} \right\} \quad (8)$$

Prior distribution

To make Bayesian inference for the unknown β_j, one must choose from two types of prior (informative or non-informative) distributions. The vital step in Bayesian inference is how to choose the prior distributions. When there is something known about the values of the unknown parameters (β_j), the informative prior distributions are employed. However, if there is nothing or little is known about the unknown parameters, or sometimes one wants to make sure that prior information does not play a big role in the analysis (that is, the data is allowed to be influential in the analysis), then non-informative priors are applied (this is sometimes known as objective-Bayesian analysis). In this paper, the researchers chose non-informative priors for the regression coefficient, or variances of random effects, because they had no prior knowledge about the parameters. In many Bayesians software, including R software, a default normal non-informative prior with no specific information provided is chosen, making them a common choice for general-purpose modelling. This study used a default flat prior Normal (Gaussian) distribution to estimate regression coefficients. i.e $\beta_j \sim N(\mu_j, \sigma_j{}^2)$. This prior distribution is the simplest over the other priors (Laplace, Cauchy, etc). The Gaussian prior distribution is given by:

$$P(\beta_j | \mu_j, \sigma_j{}^2) = \frac{1}{\sqrt{2\pi\sigma_j{}^2}} exp\left[-\frac{1}{2\sigma_j{}^2} (\beta_j - \mu_j)^2 \right] \quad (9)$$

In this paper, the default values for hyper-parameters $\mu_j, \sigma_j{}^2$ are chosen as $\mu = 0$, and $\sigma = 1000$ (large enough) such that they give non-informative priors.

Posterior distribution

The Bayes theorem allows a combination of the likelihood of data and prior beliefs about the model parameters. Bayesian inference derives posterior probability distributions by

multiplying the full likelihood function by the prior distribution. The posterior distribution of the unknown parameters β_j for the BLR with Normal prior distribution is:

$$posterior = \prod_{i=1}^{n} \left\{ \left[\frac{exp(\beta_0+\beta_1 x_{1i}+\beta_2 x_{2i}+\cdots+\beta_p x_{pi})}{1+exp(\beta_0+\beta_1 x_{1i}+\beta_2 x_{2i}+\cdots+\beta_p x_{pi})} \right]^{y_i} \left[1 - \frac{exp(\beta_0+\beta_1 x_{1i}+\beta_2 x_{2i}+\cdots+\beta_p x_{pi})}{1+exp(\beta_0+\beta_1 x_{1i}+\beta_2 x_{2i}+\cdots+\beta_p x_{pi})} \right]^{1-y_i} \right\} \times$$

$$\left\{ \prod_{j=0}^{p} \frac{1}{\sqrt{2\pi\sigma_j{}^2}} exp\left[-\frac{1}{2\sigma_j{}^2} \left(\beta_j - \mu_j\right)^2 \right] \right\} \tag{10}$$

Markov Chain Monte Carlo (MCMC) algorithm

Due to the complexity of the posterior distribution in (10), it cannot be evaluated analytically. The MCMC methods can be used to estimate the parameters. There are three basic MCMC algorithms that can be used, namely: the *Gibbs sampler algorithm*, the *Metropolis algorithm* and the *Metropolis-Hastings (MH) algorithm*. In this paper, the researchers used the MH algorithm to help generate random samples from the underlying posterior distribution. To solve and estimate the marginal posterior distributions for this model's parameters, the basic steps of this algorithm are as follows:

Step 1: Take the initial value for the parameter: $\theta_{j=0} = \theta^* \sim p\left(\theta | \theta_{j-1}\right)$. The starting values can be obtained via MLE.

Step 2: Generate a random sample from a uniform distribution $U(0,1)$.

Step 3: Compute the ratio $R = \frac{p(\theta^*|X,y)p(\theta_{j-1}|\theta^*)}{p(\theta_{j-1}|X,y)p(\theta^*|\theta_{j-1})}$

Step 4: Compare R with a $U(0,1)$ random draw u. If $R > u$, then set $\theta_j = \theta^*$. However, if $R < u$, set $\theta_j = \theta_{j-1}$

Step 5: Set $j = j + 1$ and repeat steps 1 to 4 until enough draws are obtained.

The reader can follow the steps of this algorithm in Chen et al. (2007)[60].

Supplementary Materials B

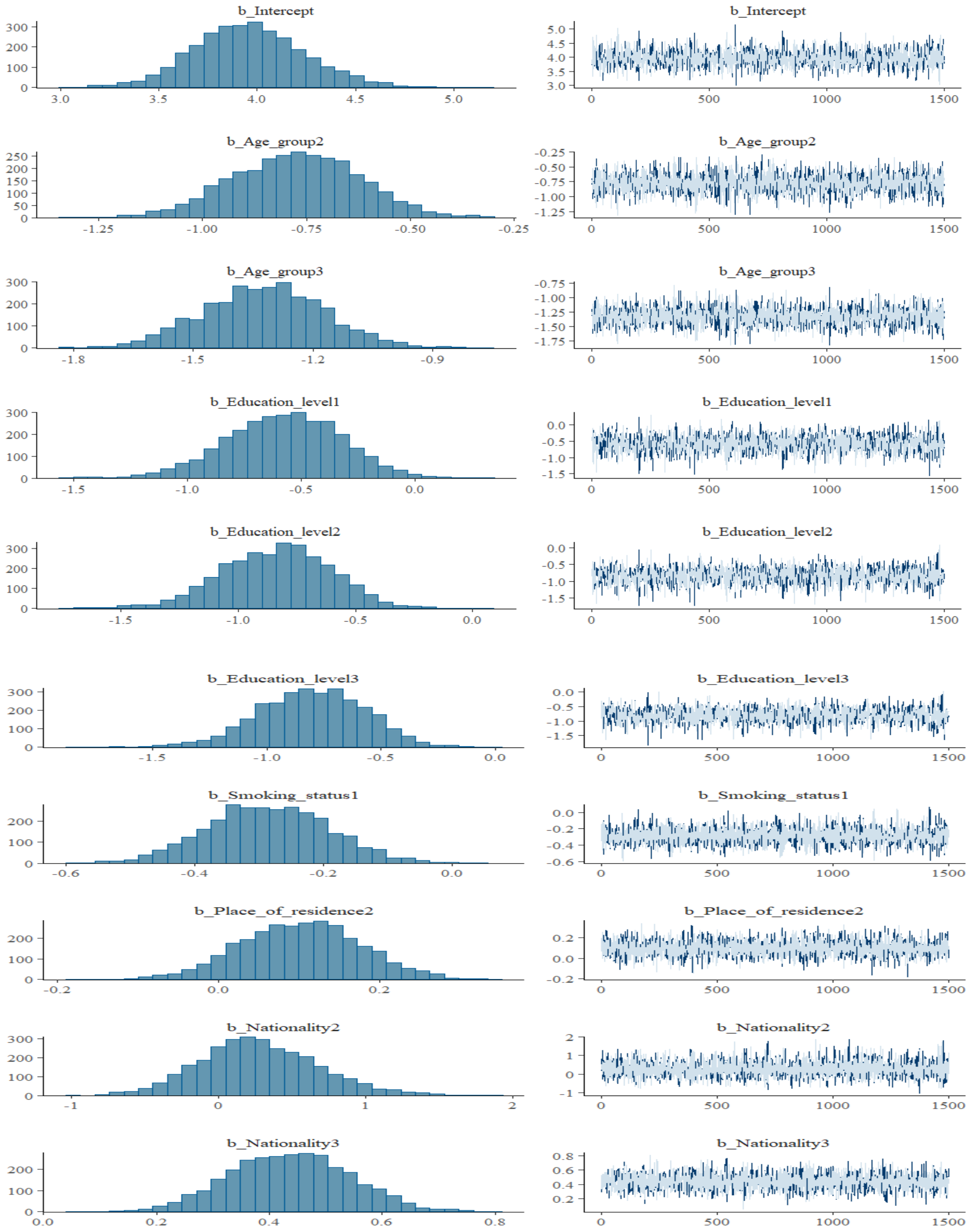

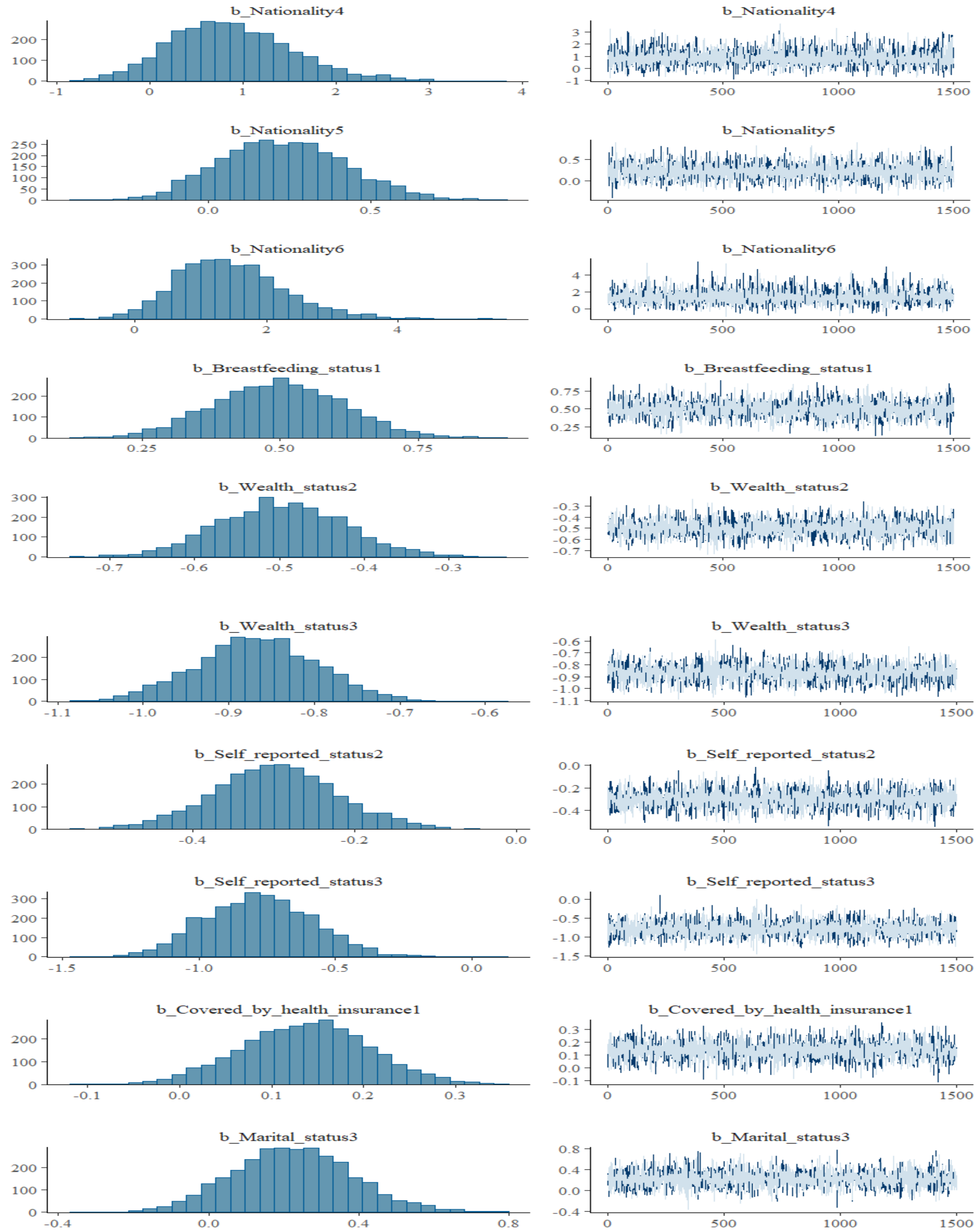
b_Nationality4
b_Nationality4
b_Nationality5
b_Nationality5
b_Nationality6
b_Nationality6
b_Breastfeeding_status1
b_Breastfeeding_status1
b_Wealth_status2
b_Wealth_status2
b_Wealth_status3
b_Wealth_status3
b_Self_reported_status2
b_Self_reported_status2
b_Self_reported_status3
b_Self_reported_status3
b_Covered_by_health_insurance1
b_Covered_by_health_insurance1
b_Marital_status3
b_Marital_status3

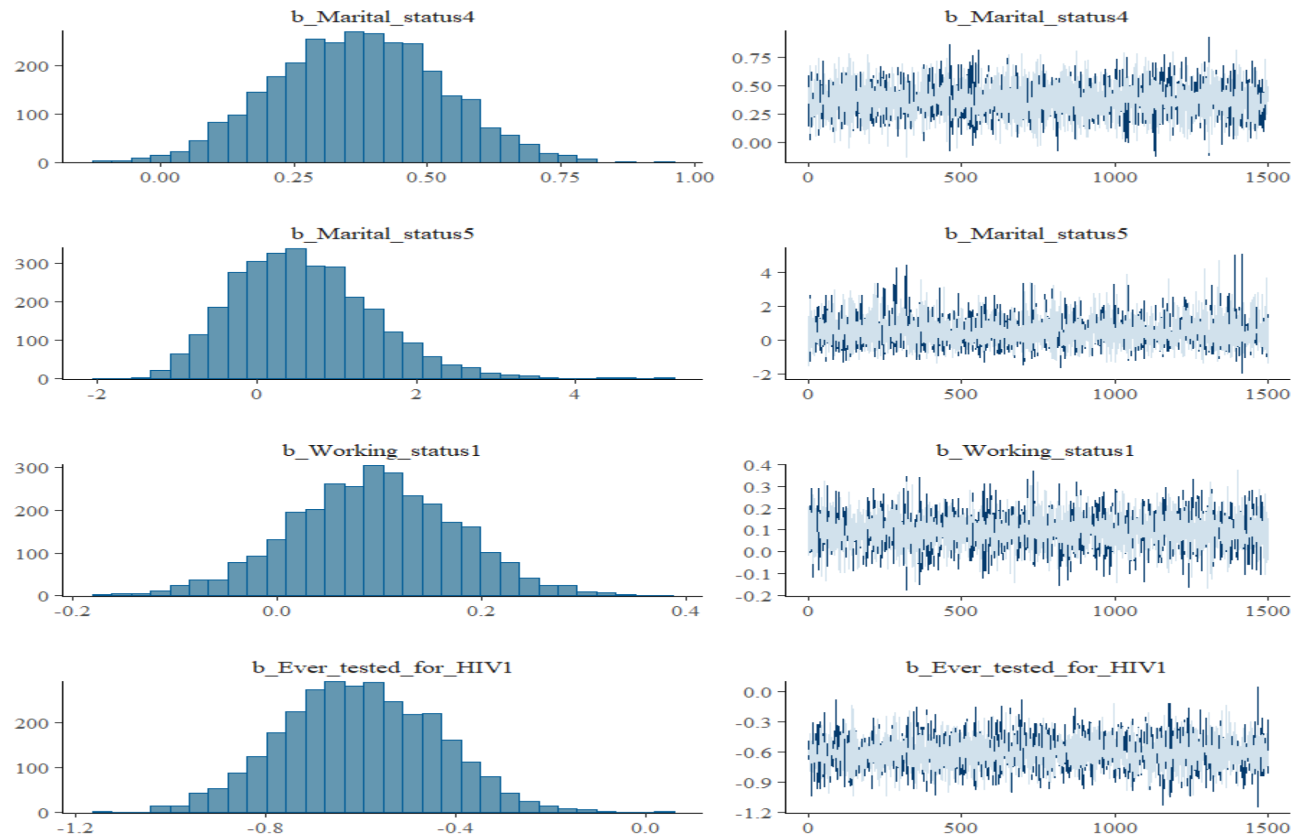

Figure S1: Posterior distribution for the parameters of the Bayesian logistic regression under the Gaussian prior distributions. Right panels are posterior densities (histogram) and left panels give the MCMC trace plots for each parameter.

Reference

60. Chen T, Morris J, Martin E. Gaussian process regression for multivariate spectroscopic calibration. Chemometrics and Intelligent Laboratory Systems. 2007 May 15;87(1):59-71.
